# Medical Students Respond: Question Precision and Gender Differentiation. Comment on “Understanding Medical Students’ Attitudes Toward Learning eHealth: Questionnaire Study”

**DOI:** 10.2196/24993

**Published:** 2021-02-11

**Authors:** Ahmad Almohtadi, Minh Van, Golnoush Seyedzenouzi

**Affiliations:** 1 St George's University of London London United Kingdom

**Keywords:** eHealth, medical students, medical education

We read with great interest the article by Vossen et al [1] investigating the preparedness of medical students to take advantage of eHealth innovations in medicine and their attitude toward its implementation in medical education. The successful utilization of eHealth has never been more relevant than it is today, allowing for better workflows, fewer errors, scalability of record-keeping, and, importantly, remote consultations to reduce the transmission and risk of COVID-19 [2]. Therefore, this article comes at a critical time to allow for a better understanding of the factors affecting the willingness of medical students to interact with eHealth to allow for meaningful change to be implemented.

Although the authors have attempted to investigate the attitudes of students toward learning eHealth, the questions used provide a vague description of said eHealth. For example, the authors ask, “I feel prepared to take advantage of the technological developments within the medical field” without describing the nature of the technological options available. eHealth in clinical practice can vary from simple telecommunication consultations to complex diagnostic artificial intelligence; thus, as medical students, we would expect the questions to be more focused in order to successfully judge our preparedness and consequently contribute to more reliable and practical results. In their study, Walpole et al [3] described the specifics of eHealth in their questionnaire, for example, “use of computers and other information systems, including storing and retrieving information,” allowing students to accurately respond to the questions, thereby producing reliable results. By describing the eHealth measure, there is less room for interpretation, which allows the results to be generalized to the general medical student population.

Additionally, responders to the survey were mainly female medical students (215/303, 71.0%), which may have skewed the results of this study. A similar study by Haluza et al [4] exploring eHealth behavior and gender demonstrated that females are more likely to engage in health technology. In the study, 89.6% of the females engaged in online health-related services compared to 77.8% of males, highlighting that there is a gender difference among eHealth users. Addressing this discrepancy would therefore produce reliable results that can be used to implement eHealth and telemedicine strategies that would promote digital skill use in medical practice. The role of gender needs to be assessed in a more extensive survey that would better represent the cohort.

Even though data regarding the technical skill level of participants were collected, the authors did not elaborate on the link between technical literacy and medical students’ attitude toward implementing eHealth into their future work environment. Previous experience with technology can impact one’s likelihood of taking advantage of eHealth in clinical practice. This was evident in a study by Olok et al [5], which showed that the level of ICT (information and communications technology) skill was a significant predictor of eHealth use among medical professionals. Therefore, understanding this association can guide future interventions to target specific groups of medical students to promote their engagement with eHealth.

We congratulate the authors on this research as it provides important insights into student doctors’ attitudes toward eHealth. However, we recommend that the authors use a more descriptive set of questions, as well as adjust for the discrepancies in gender. This would allow for representative results that can be used to influence change in eHealth medical school curricula.

## Abbreviations

ICT: information and communications technology
